# Beneficial effect of *Mentha suaveolens *essential oil in the treatment of vaginal candidiasis assessed by real-time monitoring of infection

**DOI:** 10.1186/1472-6882-11-18

**Published:** 2011-02-28

**Authors:** Donatella Pietrella, Letizia Angiolella, Elisabetta Vavala, Anna Rachini, Francesca Mondello, Rino Ragno, Francesco Bistoni, Anna Vecchiarelli

**Affiliations:** 1 Microbiology Section, Department of Experimental Medicine and Biochemical Sciences, University of Perugia, Via del Giochetto, 06122 Perugia, Italy; 2 Department of Public Health and Infectious Diseases, University of Rome 'La Sapienza', Piazzale Aldo Moro, 00185 Rome, Italy; 3 Department of Infectious, Parasitic and Immune-mediated Diseases, Istituto Superiore di Sanità, Viale Regina Elena 299, 00161 Rome, Italy; 4 Department of Chemistry and Drug Technologies, University of Rome 'La Sapienza', Piazzale Aldo Moro, 00161 Rome, Italy

## Abstract

**Background:**

Vaginal candidiasis is a frequent and common distressing disease affecting up to 75% of the women of fertile age; most of these women have recurrent episodes. Essential oils from aromatic plants have been shown to have antimicrobial and antifungal activities. This study was aimed at assessing the anti-fungal activity of essential oil from *Mentha suaveolens *(EOMS) in an experimental infection of vaginal candidiasis.

**Methods:**

The *in vitro *and *in vivo *activity of EOMS was assessed. The *in vitro *activity was evaluated under standard CLSI methods, and the *in vivo *analysis was carried out by exploiting a novel, non-invasive model of vaginal candidiasis in mice based on an *in vivo *imaging technique.

Differences between essential oil treated and saline treated mice were evaluated by the non-parametric Mann-Whitney U-test. Viable count data from a time kill assay and yeast and hyphae survival test were compared using the Student's t-test (two-tailed).

**Results:**

Our main findings were: i) EOMS shows potent candidastatic and candidacidal activity in an *in vitro *experimental system; ii) EOMS gives a degree of protection against vaginal candidiasis in an *in vivo *experimental system.

**Conclusions:**

This study shows for the first time that the essential oil of a Moroccan plant *Mentha suaveolens *is candidastatic and candidacidal *in vitro*, and has a degree of anticandidal activity in a model of vaginal infection, as demonstrated in an *in vivo *monitoring imaging system. We conclude that our findings lay the ground for further, more extensive investigations to identify the active EOMS component(s), promising in the therapeutically problematic setting of chronic vaginal candidiasis in humans.

## Background

*Candida albicans *is a major fungal pathogen of humans [1,2] and a commensal organism of the gastrointestinal tract. In severely immunocompromised patients this fungus causes high morbidity and mortality. *C. albicans *is also the etiological agent of vulvovaginal candidiasis, a common pathological condition, afflicting normal women of fertile age, which frequently develops into a chronic, substantially incurable, disease [3].

Different classes of antimycotic drugs are available to treat fungal infections. The azoles, particularly fluconazole, remain among the most common antifungal drugs, but their intensive clinical use for both therapy and prophylaxis has favoured the emergence of resistant strains [4]. The phenomenon of drug resistance has raised interest in substances of natural origin as a therapeutic alternative. Essential oils (EO) of aromatic plants are used by companies for the production of soaps, perfumes and toiletries. Many of them are also used in traditional medicine for various purposes [5-7]. In the last years various EO have been found to show antimicrobial, antioxidant anticancer and other pharmacological activities [8-10]. Particularly, a number of EO have been tested for *in vivo *and *in vitro *antimycotic activity and some have been shown to be potential antifungal agents.

The EO have a complex composition based on a number of constituents with low molecular weight, and their biological activities are due either to a main component of the mixture, usually a monoterpene, or to the synergic action of multiple compounds [11].

*Mentha suaveolens *has been used in the traditional medicine of Mediterranean areas and has a wide range of effects: tonic, stimulating, stomachic, carminative, analgesic, choleretic, antispasmodic, sedative, hypotensive and insecticidal. It shows depressor activity, analgesic and antiinflammatory action [12].

*Mentha suaveolens *plants collected in various regions of Morocco contains a high percentage of oxides such as piperitenone oxide (PEO) and piperitone oxide (PO), terpenic alcohol (fenohol, p-cymen-8-ol, geraniol, terpineol and borneol) and terpenic ketones (pulegone and piperitenone) all of which account for 65% to 90% of the total essential oil. The antimicrobial activity of PO, even if generally comparable to that of PEO, seems to be two-fold lower than that of PEO against yeast [13]. No studies have however addressed the *in vivo *activity of *Mentha suaveolens *EO in a suitable experimental model of vaginal candidiasis under controlled conditions. Thus, in this study we have tested the *in vitro *and *in vivo *activity of *M. suaveolens *EO against *C. albicans*. Particularly, for *in vivo *activity, we used a recently developed, non-invasive *in vivo *imaging technique, which exploits a novel cell surface luciferase as reporter gene [14].

For both *in vitro *and *in vivo *studies, we used Jasmine Oil as a negative control and Tea Tree Oil as a positive control.

## Methods

### Essential oils

*Mentha suaveolens *essential oil was kindly provided by the Department of Chemistry and Drug Technologies, University of Rome "La Sapienza", Italy. It was obtained from wild-type plants grown in Tarquinia forests located around 60 miles from Rome. The oil was extracted by four-hour hydro distillation of the leaves using a Clevenger-type apparatus as previously described [15], then analyzed for chemical composition by gas chromatography and mass spectroscopy (DMePe BETA PS086, 0.25 mm film on a 25 m column, diameter of 0.25 mm, operating at 220°C and eluting with helium). Compounds were identified by the application of the NIST 08 Mass Spectral Library. Analysis revealed that piperitenone oxide constitutes 90% of EOMS. Limonene and 1,8-cyneole were also present, among other minor constituents.

Essential oils of tea tree (*Melaleuca alternifolia) *(TTO) and jasmine oil (*Jasminum grandiflorum*) (JO) also used in this research were commercial oils purchased form Named (Lesmo, Italy) and Erboristeria Magentina (Torino, Italy), respectively. They were obtained by steam distillation from leaves and young branches of tea tree, and from flowers of jasmine. TTO is pure, extracted without additives and was used as a positive control, because of documented antifungal activity [16,17] while jasmine oil, which was shown to be inactive against fungal growth, was used as a negative control [18].

Fluconazole was obtained from Sigma-Aldrich (Germany).

### Microorganisms

Different strains of *Candida albicans *were used in the study: four clinical isolates from AIDS patients AIDS68, AIDS6, AIDS37 and AIDS126, CO23 isolated from a subject with vulvo-vaginal candidiasis susceptible to micafungin and fluconazole and the drug-resistant strains CO23RFK (micafungin-resistant) and CO23RFLU (fluconazole-resistant) [19], CA2, an echinocandin-resistant, non-germinative strain that grows as a pure yeast form at 28-37°C in conventional mycologic media [20], GR5 isolated from a woman with recurrent vaginal candidiasis, 3153 intrinsically resistant to fluconazole, ATCC10231 and ATCC24433. *C. albicans *CA1398 carrying the *ACT1p-gLUC59 *fusion (*C. albicans *gLUC59) or *C. albicans *CA1398 that did not express *gLUC59 *(control strain) were used in the models of vaginal *Candida *infections [14]. For experimental infections, cells from stock cultures in YPD agar (1% yeast extract, 2% peptone, 2% glucose, 1.5% agar, all w/v) with 50 μg/ml chloramphenicol were grown in YPD broth (1% yeast extract, 2% peptone,2% glucose, all w/v) at room temperature for 24 h, then harvested by centrifugation, washed, counted in an haemocytometer, and resuspended to the desired concentration in sterile physiological saline. In order to examine the effect of the oil on the mycelia form of *Candida*, yeasts were grown for 4 h in RPMI 1640 plus 10% FBS at 37°C, then hyphae were washed and incubated with different concentrations of essential oils (EOMS, TTO and JO) for 24 h at 37°C. Yeasts for infection were harvested from overnight cultures in YPD agar plates and adjusted to the concentration 10^9^/ml in sterile physiological saline.

### Minimal Inhibitory Concentration (MIC) assay

The Minimal Inhibitory Concentration (MIC) was determined by micro-broth dilution method according to the Clinical and Laboratory Standards Institute/National Commitee for Clinical Laboratory Standards (CLSI/NCCLS) Approved Standard M27-A3, 2008 [21]. Fluconazole 0.5 g/L solution was prepared by dissolving the agent in endotoxin free water. Solutions of essential oils (100 g/L) were prepared in RPMI1640. Briefly, to determine the MIC of EOMS, TTO, JO or Fluconazole, RPMI-1640 supplemented with MOPS at pH 7 was used. EOMS, TTO and JO were diluted in RPMI-1640 supplemented with Tween 80 (final concentration of 0.001% v/v). The dilutions, ranging from 0.01219 to 12.48 g/L of the essential oils, were prepared in 96 well plates. The inoculum size was about 2.5 × 10^3^cells/ml. The plates were incubated at 30°C for 24-48 h. To determine the hyphae survival, *C. albicans *cells were first grown for 4 h in RPMI supplemented with 10% of FBS serum and then treated with different essential oils.

### Minimal Fungicidal Concentration (MFC) assay

The Minimal Fungicidal Concentration (MFC) was determined as the lowest concentration of Fluconazole or essential oils at which no microbial growth was observed. For the MFC determination, Sabouraud dextrose agar plates were seeded with 10 μl of cell suspensions taken from the wells of the plates of MIC assay where cell growth was not observed. These plates were incubated at 30°C for 24-48 h and colony forming units (CFU) growth was evaluated.

### Time killing

To confirm the fungicidal activity of EOMS, time-kill procedures were performed as described by Klepser [22]. Cells sub-cultured in YPD at 28°C for 24 h were centrifuged, washed and resuspended at a concentration of 2.5 × 10^5^cell/ml in RPMI supplemented with EOMS or TTO and incubated at 28°C. Essential oil concentrations used in the test were equivalent to 1, 2, 4, and 8 times the MIC. At predetermined time points (0, 0.5, 1, 2, 4, 6, 8, 24 and 48 hours) of incubation, 100 μl aliquots were removed from the test solution and tenfold serial dilutions were performed. 100 μl aliquot from each dilution was spread on the surface of Sabouraud dextrose agar plates and incubated at 37°C for 48 h for determination of CFU/ml.

### Cell lines

Monomac-6, a human tumour cell line which was initially obtained from peripheral blood of a 60-year-old man with acute monocytic leukaemia, and L929, a fibroblast-like cell line cloned from strain L (the parent strain was derived from normal subartaneous areolar and adipose tissue of a male C3H/An mouse) were grown in a humidified atmosphere containing 5% of CO_2 _at 37°C. The culture medium consisted of RPMI 1640 with glutamine, 10% FBS (foetal bovine serum) and antibiotics. Every three or four days the cultures were split.

### Cytotoxicity assay

The cytotoxicity was tested by the determination of the cell ATP level by ViaLight^® ^Plus Kit (Lonza). The method is based upon the bioluminescent measurement of ATP that is present in all metabolically active cells. The bioluminescent method utilizes an enzyme, luciferase, which catalyses the formation of light from ATP and luciferin. The emitted light intensity is linearly related to the ATP concentration and is measured using a luminometer. To perform cytotoxicity tests, cells were recovered and counted and adjusted to the concentration 10^6^/ml. The examinations were carried out for essential oils (EOMS, TTO and JO) and the control (cells not treated). Various 1:2 dilutions of the above mentioned oils were prepared in the medium (RMPI 1640, 10% FBS, antibiotics) in order to achieve final concentrations in the wells: 1000-500-250-125-62.5-31-16-8-4-2-1-0 mg/L. Each concentration was tested in triplicate. After adding oils into appropriate wells, cells were added to each well to obtain the concentration of 10^5^cells/well and incubated for 2 h at 37°C. Plates were left in a room temperature to cool for 10 minutes and then the Cell Lysis Reagent was added to each well to extract ATP form the cells. Next, after 10 minutes the AMR Plus (ATP Monitoring Reagent Plus) was added and after 2 more minutes the luminescence was read using a microplate luminometer (TECAN).

### Mice

Female CD1 mice obtained from Harlan Italy Laboratories (Udine, Italy) were used at 4 to 6 weeks of age. Mice were allowed to rest for 1 week before the experiment; by that time the animals were roughly 5 to 7 weeks old. Animals were used under specific-pathogen-free conditions that included testing sentinels for unwanted infections; according to the Federation of European Laboratory Animal Science Association standards, no infections were detected.

The experimental research was approved on 25 January 2008 by the Ethics Committee of the University of Perugia.

### Infection and treatment

Mice infection was performed as previously described with minor adaptations [23]. Mice were maintained under pseudoestrus condition by subcutaneous injection of 0.2 mg of estradiol valerate in 100 μl of sesame oil (Sigma-Aldrich) 6 days prior to infection and weekly until the completion of the study. Mice anaesthetized with 2.5-3.5 (v/v) isofluorane gas were infected twice at a 24 h interval with 10 μl of 10^9 ^cell/ml of *C. albicans *gLUC59 or the control strain. Cell suspensions were administered from a mechanical pipette into the vaginal lumen, close to the cervix. To favour vaginal contact and adsorption of fungal cells, mice were held head down for 1 min following inoculation. Mice were then allowed to recover for 24-48 h, during which the *Candida *infection was established.

The intravaginal treatment with TTO, EOMS and JO (500 μg/10 μl/mouse) was begun 2 h before the first challenge and then it was repeated every two days until day +21.

### Monitoring of mouse vaginal infection

To monitor the infection during the treatment with essential oil, every day post-infection (starting 48 h after challenge) 10 μl (1 mg/ml in 1:4 methanol:H_2_O) of coelenterazine was added to the vaginal lumen. Afterwards, mice were imaged in the IVIS-200TM imaging system under anaesthesia with 2.5% isoflurane. Total photon emission from vaginal areas within the images (Region Of Interest, ROI) of each mouse was quantified with Living ImageR software package. In selected experiments mice were anaesthetized with 2.5% isoflurane and then held head down, the vaginal lumen was thoroughly washed with 150 μl of saline. To determine the fungal load in the vagina, 50 μl of the lavage fluids from each mouse were plated on YPD agar plus chloramphenicol (50 μg/ml), then CFUs were evaluated.

### Statistical analysis

Differences between essential oil treated and saline treated mice were evaluated by the non-parametric Mann-Whitney U-test. Viable count data from time kill assay and yeast and hyphae survival test were compared using the Student's t-test (two-tailed). *P*-values of < 0.05 were considered significant.

## Results

### MIC, MFC and Killing Kinetics

The initial determination of the antifungal activity of essential oils (EOMS, TTO and JO) was performed *in vitro *by standardized CLSI/NCCLS methods[21] and this was done against all strains of *C. albicans *used throughout this study. MIC values fell in a range of 0.39-0.78 g/L for EOMS and 0.78-3.12 g/L for TTO. The MFC values ranged from 0.39-1.56 g/L for EOMS and 1.56-6.24 for TTO, thus very close to MIC values. In our experimental system, TTO was less efficient than EOMS especially when the oils were tested against fluconazole resistant strains. In addition MFC values for TTO were higher than EOMS for all strains tested (table 1). JO, used as a negative control, did not affect the growth of any strain tested.

**Table 1:** Antifungal activity of EOMS, TTO, JO and fluconazole on different *Candida albicans *strains.

	EOMS		TTO		JO		Fluconazole	
**Strains**	**MIC g/L**	**MFC g/L**	**MIC g/L**	**MFC g/L**	**MIC g/L**	**MFC g/L**	**MIC mg/L**	**MFC mg/L**

**AIDS68**	0.39	0.78	3.12	3.12	> 100	> 100	> 64	> 128

**AIDS6**	0.39	0.78	3.12	6.24	> 100	> 100	> 64	> 128

**AIDS37**	0.39	0.39	3.12	6.24	> 100	> 100	> 64	> 128

**AIDS126**	0.39	0.78	3.12	6.24	> 100	> 100	0,25	2

**CA2**	0.39	0.39	3.12	6.24	> 100	> 100	0,5	> 128

**GR5**	0.39	0.78	3.12	3.12	> 100	> 100	2	> 128

**3153**	0.39	0.78	3.12	6.24	> 100	> 100	64	> 128

**CO23**	0.39	0.39	0.78	1.56	> 100	> 100	0.25	1

**CO23RFK**	0.78	1.56	0.78	1.56	> 100	> 100	0.25	1

**CO23RFLU**	0.78	1.56	0.78	3.12	> 100	> 100	> 64	> 128

**ATCC10231**	0.39	0.39	3.12	6.24	> 100	> 100	4	4

**ATCC24433**	0.39	0.39	3.12	6.24	> 100	> 100	0.25	1

**gLUC59**	0.39	1.56	3.12	6.24	> 100	> 100	0.39	0.78

**gLUC59 Control strain**	0.39	1.56	3.12	6.24	> 100	> 100	0.39	0.78

**Figure 1:**
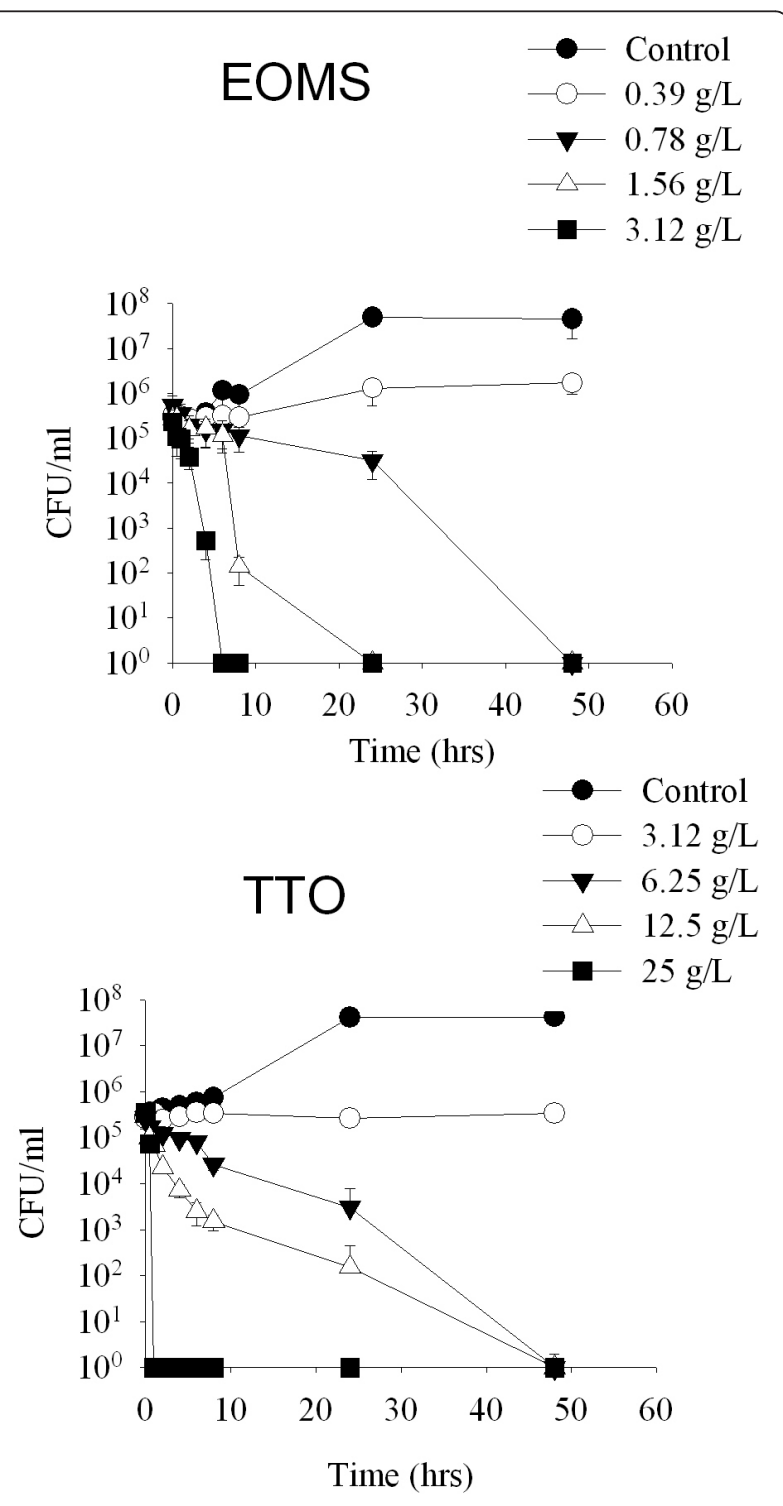
**Time kill curve of EOMS and TTO against *Candida albicans *gLUC59**. Cells were untreated (control) or treated with 0.39, 0.78, 1.56, or 3.12 g/L of EOMS or treated with 3.12, 6.25, 12.5 or 25 g/L of TTO for different time periods (0, 0.5, 1, 2, 4, 6, 8, 24 and 48 hours). After incubation survival cells were determined by cultivation on Sabouraud dextrose agar plates at 37°C for 48 h. Results are expressed as CFU/ml and indicated as mean ± SEM of triplicate samples. Data are representative of one of three independent experiments. The statistical significance was evaluated with the Student's t-test (two-tailed). *P-values of < 0.05 were considered significant.

To obtain more insight into the anticandidal activity of EOMS on gLUC59, i.e the strain used in the experimental vaginal infection (see below), a time-kill test at concentrations equivalent to 1, 2, 4, and 8 times of the MIC value (0.39 g/L) was performed. As reported in Figure 1, at a concentration of 2 × MIC (0.78 g/L), the number of colonies was significantly reduced after 24 hrs of incubation (*P *< 0.05) and the total fungicidal effect was observed within 48 hrs of contact. The results demonstrated that *C. albicans *gLUC59 was highly susceptible to EOMS. In parallel we analyzed the time kill test for TTO, confirming that this oil exerted a fungicidal effect within 48 hrs at a concentration higher (3,12 g/L; *P *< 0.05) than that observed with the EOMS (0,78 g/L).

### Yeasts and the mycelial form have different susceptibilities to EOMS

**Figure 2:**
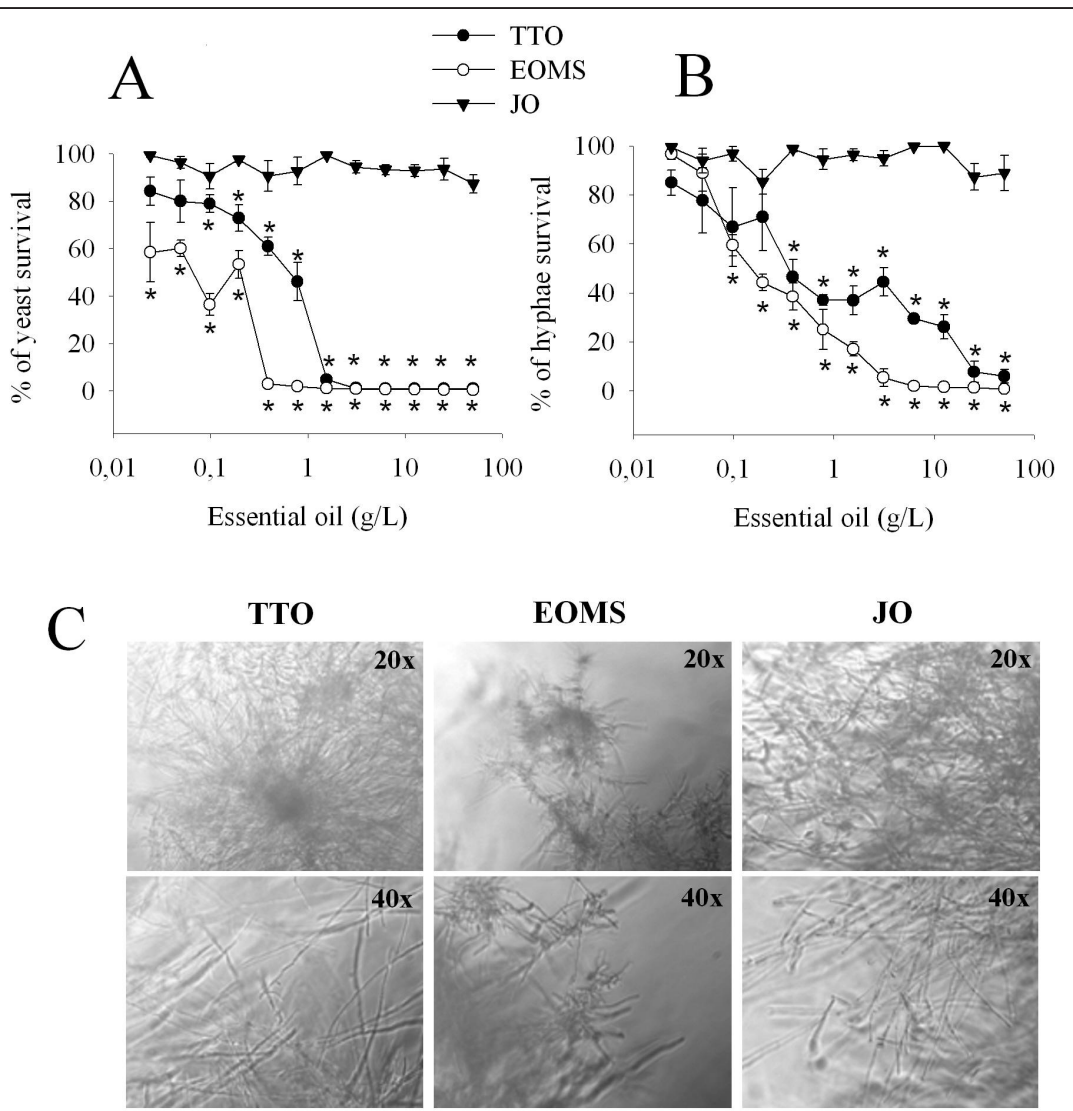
**Effect of essential oil on yeast and hyphae survival**. gLUC59 *Candida albicans *yeast cells (A) and preformed hyphae (B) were treated with different concentrations of essential oils (EOMS, TTO and JO) for 24 h. After incubation 10 μl of coelenterazine substrate (1 mg/ml) was added to each well and samples were read using a luminometer. Results are expressed as percentage of yeast or hyphae survival and indicated as mean ± SD of triplicate samples are from one of three experiments with similar results. The statistical significance was evaluated with the Student's t-test (two-tailed). *P-values of < 0.05 were considered significant. The effect on preformed hyphae was microscopically examined after 24 h of treatment with essential oil (C). Original magnification of 20x or 40x is indicated in the micrographs. The results are representative of one of three independent experiments.

Given that EOMS affects *C. albicans *yeast forms of growth, we extended our investigations to the virulent mycelial form of *C. albicans*. To this end *C. albicans *was cultured at 37°C for 4 h in the presence of 10% of FBS serum. Microscopic examination demonstrated >90% mycelial conversion under our conditions. As shown in Figure 2, hyphal forms were less susceptible to EOMS than yeast forms, since inhibition of hyphal growth is obtained at a significantly higher concentration (0.098 g/L) than the concentration required to inhibit yeast forms of growth. In fact, a concentration of 0.5 g/L was able to completely inhibit the yeast cells but not the hyphal form (Figure 2A-B) Notably, the EOMS showed a greater inhibitory effect than TTO, in both yeast cells (0.05 vs 0.098 g/L respectively) and the hyphal form (0.098 vs 0.39 g/L respectively). Jasmine Oil did not affect the viability of yeast or mycelial cells. The lack of effect of EOMS 0.5 g/L on hyphal survival was documented by microscopic examination of hyphal damage observed after addition of EOMS (Figure 2C).

**Figure 3:**
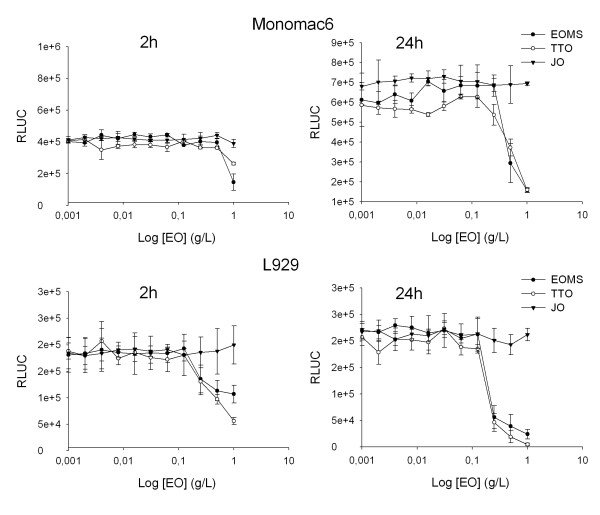
**Cytotoxicity of essential oils on mammalian cells**. Monomac 6 and L929 cells were treated with different concentrations of essential oils for 2 h. The cytotoxicity was tested by the determination of the cell ATP level by a bioluminescent method. Results, expressed as Relative luciferase activity (RLUC), represent the mean ± SD of three different experiments. The statistical significance was evaluated with the Student's t-test (two-tailed). *P-values of < 0.05 were considered significant.

Experiments were also performed to examine whether the EOMS expressed cytotoxicity towards immune cells. To this end, Monomac 6 and L929 cell lines were treated with various concentrations of EOMS for 2 and 24 h. The results reported in Figure 3 show that high concentrations of EOMS (0.5 and 1 g/L), i.e. higher than the MIC value, were necessary to exert cytotoxicity on Monomac 6 and L929 cell lines. Using the same concentrations a similar trend was observed for TTO, while no cytotoxicity resulted from JO treatment.

### Effect of EOMS on vaginal candidiasis

Given the encouraging results observed *in vitro*, we wondered whether these beneficial effects against *C. albicans *could be reproduced in an *in vivo *system. To this purpose we exploited the new *in vivo *imaging technique which we have recently developed in our laboratory [14,24] to assess therapeutic efficacy in an estrogen-dependent mouse model of vaginal candidiasis.

**Figure 4:**
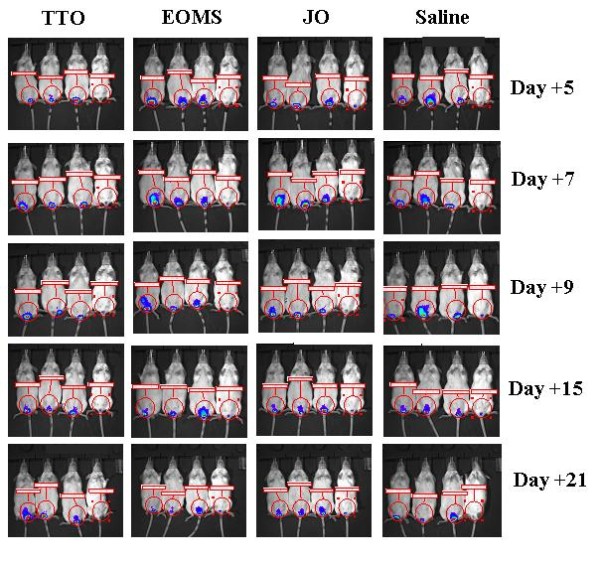
***In vivo *imaging of mice vaginally infected with *Candida albicans *and treated with essential oils**. The vaginal lumen of mice under pseudoestrus condition were infected for 2 consecutive days with 10 μl of a 10^9 ^cell/ml suspension of *Candida albicans *gLUC59 (first three mice of each group) or control strain (fourth mouse of each group) and treated with the different essential oils 2 h before the first challenge and then every two days. After 5, 7, 9, 15 and 21 days post-infection mice were treated intravaginally with 10 μg of coelenterazine and imaged in the IVIS-200TM imaging system under anaesthesia with 2.5% isoflurane. Total photon emission from vaginal areas within the images (Region Of Interest, ROI) of each mouse was quantified with Living ImageR software package. Data are from one of three experiments with similar results.

**Figure 5:**
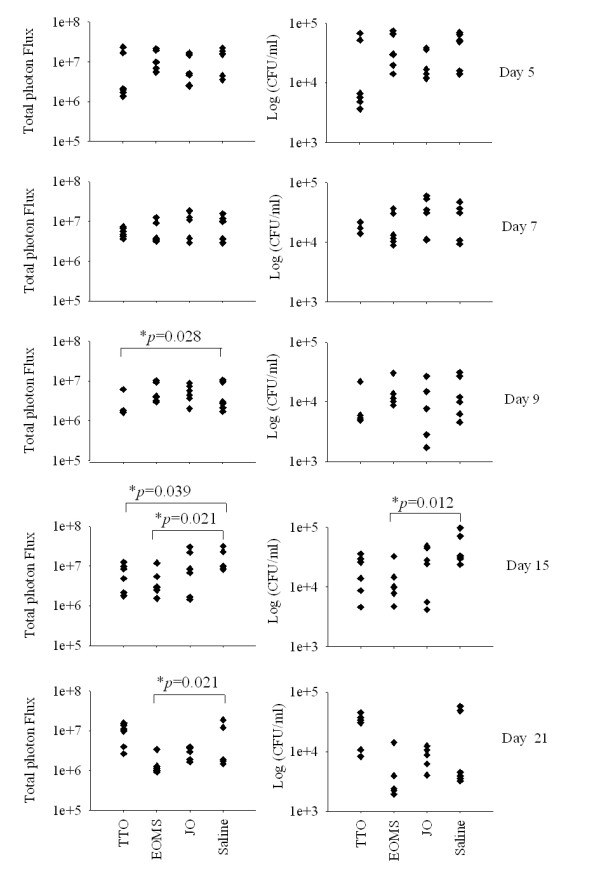
**Measurement of *Candida albicans *load in mice treated with different essential oils**. The vaginal lumen of mice under pseudoestrus condition were infected with *Candida albicans *gLUC59 and then treated with the essential oils 2 h before the first challenge and then every two days. After 5, 7, 9, 15 and 21 days post-infection 6 mice per group were anaesthetized, imaged in the IVIS-200TM imaging system, and the vaginal lumen was thoroughly washed with 150 μl of saline using a mechanical pipette. The fungal burden of vaginal lavage fluids was determined by evaluating the colony forming units (CFU) assay. For CFU assay 50 μl of the lavage fluids were diluted and seeded in YPD agar plus chloramphenicol. Results were reported and the statistical significance was evaluated with the non-parametric Mann-Whitney U-test. *P < 0.05 (essential oil treated mice versus saline treated mice).

Estradiol treated mice were infected with *C. albicans *expressing luciferase gene gLUC and EOMS, TTO or JO were administered intravaginally 2 h before the first challenge and then every two days. The course of the infection was monitored at various days post challenge by *in vivo *imaging (Figure 4). The fungal load in the vagina was quantified as photon emission as well as CFU from vaginal fluids. The results reported in Figure 5 show that there is a significant reduction of *Candida *load in mice treated with EOMS with respect to diluent (saline) treated mice starting from 15 days post infection, and this beneficial effect was maintained until 21 days post infection. In this model, and under the conditions tested, TTO was only minimally effective in causing a significant reduction of vaginal fungus load, measured as photon emission at 9 and 15 days. No effect was recorded after 21 days of infection.

## Discussion

Human pathogenic fungi represent a significant proportion of the infectious agents affecting the immunocompromised host. The therapeutic options for these patients are hampered by i) the relative scarcity of active and safe antifungal drugs, most of which are essentially fungistatic rather than fungicidal, ii) antifungal drug resistance to the most active and widely used azole compounds, iii) the difficulties of devising and/or constantly maintaining effective infection control measures in the health care institutions. Overall, fungal infections in immunodepressed subjects are a very challenging problem for the health system.

Thus there is a clear demand for finding a new therapeutic approach in this era of increasing spreading of antimicrobial drug resistance and re-emergence of infectious diseases [25,26].

Recently the use of TTO as a new approach in antifungal therapy has been proposed. This natural compound appears to be effective *in vitro *against multidrug resistant *Candida *and *in vivo *against mucosal candidiasis [27]. Moreover it has also been documented that terpinen-4-ol rather than 1,8-cineole is the most likely mediator of TTO activity or, at least, a main contributor to anti-*Candida *activity [16]. In this study we used TTO as a positive control in our *in vitro *and *in vivo *experimental system.

Regarding the antimicrobial properties of EOMS recent evidence attributes larvicidal activity to this essential oil and its active compound [28]. Other important activities of EOMS include protective effects against hydrogen-peroxide-induced-cytotoxicity. Anti-*Candida *activity has been described for *Mentha piperita *[29]. Furthermore EOMS was effective against Gram positive and Gram negative microorganisms and fungi [13]. The main microbicidal components of EOMS were pulegone and piperitone oxide.

In this study we demonstrated for the first time that EOMS is endowed with potent anticandidal activity *in vitro*, both against azole-susceptible and azole-resistant *Candida *strains. In addition, EOMS was shown to be not only an inhibitor of *Candida *growth, but also able to actually kill the yeasts. We determined the time killing curves, and so discovered that EOMS was apparently more effective than the more extensively investigated TTO. All experiments were performed against a control, the jasmine oil, which proved totally ineffective.

The antifungal activity is manifested against both yeast and the mycelial form, although higher EOMS concentrations were required to kill these latter forms of growth. Finally, we provide evidence that intravaginal administration of EOMS *in vivo *is also efficacious to some degree.

For the *in vivo *assay, a stringent and controlled model of vaginal infection of mice was used. This exploits a novel cell surface luciferase as reporter gene, constructed by fusing a synthetic, codon-optimized version of the *Gaussia princeps *luciferase gene to *Candida albicans *PGA59, which encodes a glycosylphosphatidyl inositol-linked cell wall protein [14]. This technique allows a continuous, non invasive monitoring of the spatial and temporal progression of vaginal infection in a small number of live mice. The model proved useful in assaying for anticandidal protection in actively or passively immunized animals [24]. The method was paralleled by a more traditional determination of vaginal fungus load in the vagina by CFU. The in vivo imaging technique resulted much more sensitive than the classic CFU method for at least two different reasons: 1) the vaginal wash doesn't completely clear the vaginal lumen because the *Candida *hyphae are well attached to the tissue; 2) several hyphae often grew as a single colony, causing an underestimation of the fungal load.

Overall, we show here that EOMS accelerates the clearance of fungus during vaginal candidiasis, and this accelerated clearance of *Candida *is demonstrated by both photon emission and CFU measurements. The EOMS activity in our model seems superior, at least after 21 days of infection, to that of TTO, which has previously been found particularly efficacious in a rat model of vaginal candidiasis [16].

Our data are potentially relevant in the treatment of *Candida *vulvovaginal infection (VVC). This is a frequent and commonly distressing disease affecting 70-75% of childbearing age women worldwide at least once during their lives. Predisposing factors for developing an acute form of vaginal candidiasis include antibiotic and oral contraceptive usage, hormone replacement therapy, pregnancy, uncontrolled diabetes mellitus and African American ethnicity [30,31]. 5% and possibly up to 10% of women with a primary episode subsequently experience frustrating recurrent VVC (RVVC) which is defined as at least three-four specific episodes within one year [3,32].

## Conclusions

This study shows for the first time that: i) EOMS has considerable *in vitro*, candidastatic and candidacidal activity ii) EOMS administration *in vivo *accelerates the clearance of *C. albicans *during vaginal infection.

The high impact of this infection and the difficulty of finding an effective therapy reinforces the need to search for an alternative therapeutic approach to integrate or even replace the current treatment. The present results could provide the ground for further investigations, particularly aimed at identifying the therapeutically active anticandidal EOMS component(s).

## Competing interests

A patent related to piperitenone oxide, the main component of *Mentha suaveolens *essential oil, and its possible industrial application, has been filed by LA and RR.

## Authors' contributions

DP, AR carried out the *in vivo *experiments and part of the MIC evaluation. LA, EV, FM, RR carried out the essential oil extraction, the MIC and time killing curve experiments. FB participated in the design and coordination of the study. AV conceived of the study and was primarily involved in the conceptual planning of the paper. All authors read and approved the final manuscript.

## Pre-publication history

The pre-publication history for this paper can be accessed here:

http://www.biomedcentral.com/1472-6882/11/18/prepub
